# Targeting Cerebellum with Non-Invasive Transcranial Magnetic or Current Stimulation after Cerebral Hemispheric Stroke—Insights for Corticocerebellar Network Reorganization: A Comprehensive Review

**DOI:** 10.3390/healthcare10122401

**Published:** 2022-11-29

**Authors:** Eleni Aikaterini Ntakou, Grigorios Nasios, Anastasia Nousia, Vasileios Siokas, Lambros Messinis, Efthimios Dardiotis

**Affiliations:** 1 Department of Neurology, University Hospital of Larissa, Faculty of Medicine, School of Health Sciences, University of Thessaly, 41100 Larissa, Greece; 2 Department of Speech and Language Therapy, University of Ioannina, 45500 Ioannina, Greece; 3 Lab of Cognitive Neuroscience, School of Psychology, Aristotle University of Thessaloniki, 54124 Thessaloniki, Greece

**Keywords:** cerebellar stimulation, TMS, tDCS, stroke, rehabilitation

## Abstract

Non-invasive brain stimulation (NIBS) has emerged as one of the methods implemented in stroke rehabilitation. Cerebellar stimulation has gained research interest as an alternative strategy to cortical stimulation, based on the role of the cerebellum and corticocerebellar tracts in different motor and cognitive functions. This review investigates the role of the cerebellum in motor and cognitive rehabilitation following cerebral stroke using NIBS techniques combined with other therapies (e.g., speech or physical therapy). Fifteen randomized clinical trials were included. The majority of the literature findings point towards the cerebellum as a promising neurostimulation target following stroke of the cerebral cortex. Findings concern mostly rehabilitation of gait and balance, where cathodal transcranial direct current stimulation (tDCS) and intermittent theta-burst stimulation (iTBS) of the contralesional cerebellar hemisphere produce, in the presented clinical sample, improved performance and plasticity changes in the corticocerebellar network, combined with other rehabilitation methods. Data regarding aphasia rehabilitation are scarce, with right cerebellar tDCS exercising some impact in individual linguistic functions combined with language therapy. Based on recent data concerning cerebellar functions and corticocerebellar networks, along with the development of clinical protocols regarding non-invasive cerebellar (NICS) application, the cerebellum can prove a crucial intervention target in rehabilitation following stroke.

## 1. Introduction

Despite ongoing dispute on the definition of stroke, the term has been classically referred and applied to the WHO definition (1970) of ‘rapidly developed clinical signs of focal (or global) disturbance of cerebral function, lasting more than 24 h or leading to death, with no apparent cause other than of vascular origin’ [1]. It constitutes a frequent neurological disorder with major functional impact on patients, healthcare providers, and healthcare systems in general [1]. It is characterized by a variety of symptoms that extend from the motor to the cognitive domain and have the potential of leading to short- and long-term functional disability in a great percentage of patients. According to data from the World Stroke Organization, using the Disability-Adjusted Life Years parameter (DALYs), in people aged 1–69 years, 63% of healthy life is lost due to stroke-related death and disability, while in people aged 1–44 years the percentage is 18%, in all stroke types combined [2]. The burden of disability is heavier on hemorrhagic stroke, with almost 75% of healthy life years lost in the 1–69 years old group versus 50% in ischemic stroke [2]. Gait disturbances concern more than 80% of stroke survivors, with persisting disability despite rehabilitation in more than 25% of them [3]. To this day, a great number of rehabilitation strategies have been implemented, such as traditional physiotherapy and speech therapy [4], peripheral and central nerve stimulation techniques [5], robot-assisted training [6], virtual reality training [7], etc., in many cases, however recovery remains partial. Interindividual differences, the use of compensatory versus relearning techniques, patient motivation, therapy costs, and flexibility are all factors contributing to the inconsistent outcomes. Knowledge arising from the study of the circuitries that regulate motility, language, and behavior, and the neurophysiologic changes they experience due to stroke, has pointed to new directions in the rehabilitation arena, one of them being the non-invasive brain stimulation. Non-invasive brain stimulation (NIBS) refers to a field of clinical and research applications that are implemented in the neurorehabilitation domain based on their neuroplastic effect and modulation of functional circuitries and comprises mainly transcranial magnetic stimulation (TMS) and transcranial direct current stimulation (tDCS).

### 1.1. Transcranial Magnetic Stimulation (TMS)

TMS has the potential of modulating the excitability of specific brain areas using a coil that produces a rapidly alternating magnetic field, which in turn induces an electric field in the target-area. The latter is defined either by specific anatomical landmarks, or by identifying the motor ‘hot-spot’, the region where the highest motor-evoked potential is generated [8]. When current intensity exceeds a certain threshold, action potentials are evoked in target-neurons [8]. Concerning the cerebellum, coil geometry and orientation, current intensity, and depth of target-tissue influence the intended outcome. Cerebellar stimulation effects cannot be assessed directly in the cerebellum and cerebellar-brain inhibition (CBI) is used as a cerebellocortical connectivity measurement [8].

Transcranial magnetic stimulation can be applied using different protocols depending on the outcome of interest, either as a single pulse with a current intensity sufficient to produce action potentials, or as repeated stimulation (rTMS) with low (<1 Hz) or high (>5 Hz) frequency. Theta-burst stimulation (TBS) involves applying trains of 50 Hz pulses in a continuous (cTBS) or intermittent manner (iTBS). As a rule, low-frequency rTMS and cTBS cause a temporary suppression of excitation (inhibition), as indicated by decreased motor evoked potentials (MEP) amplitudes, while high-frequency rTMS and iTBS facilitate excitation, as indicated by increased MEP amplitudes [8].

### 1.2. Transcranial Direct Current Stimulation (tDCS)

tDCS creates a potential difference between two or more applied electrodes and alters the neuronal membrane potential inside the formed electrical field. Depending on the current direction (anodal or cathodal tDCS) and axonal orientation, neuronal depolarization or hyperpolarization occurs, with a general rule of increase in excitability with anodal tDCS, and decrease in excitability with cathodal tDCS [9]. tDCS has the potential of modulating neuronal activity, but not of inducing membrane potentials. Its impact depends on the physiological neuronal state and concerns the facilitation or inhibition of synaptic transmission based on synaptic plasticity mechanisms [9].

### 1.3. NIBS in Stroke Rehabilitation

An ischemic lesion can cause important changes in the complex neural networks of the lesioned tissue. The most important finding is the remapping observed in the perilesional cortex [10]. A crucial point in this metabolic cataract is the induced changes in the inter-hemispheric connections. According to the vicariation model, the unaffected hemisphere contributes to functional recovery following stroke by employing residual networks, while according to the interhemispheric competition model the natural balanced inhibition between the two hemispheres is disrupted following injury, with increased transcallosal inhibition of the affected hemisphere by the unaffected hemisphere [11]. Regarding cerebral stroke, NIBS has the potential of modulating cortical excitability in the healthy or lesioned hemisphere, aiming mainly to the restoration of disrupted intrahemispheric interactions. That is achieved by inciting through various stimulation protocols the required timing between presynaptic and postsynaptic neuronal firing, an element crucial to the development of LTP (long-term potentiation) and LTD (long-term depression) [11]. Furthermore, NIBS can modulate regions anatomically distant, but functionally related to the injured area through various corticocortical and corticosubcortical pathways [12]. Based on the theory of imbalanced inter-hemispheric inhibition, two strategies have been applied, (a) reducing excitability of contralesional M1, and (b) increasing excitability of ipsilesional M1 [13]. The first strategy concerns the use of low-frequency rTMS, cTMS, and cathodal tDCS protocols, while the second strategy concerns the use of high-frequency rTMS, iTBS, and anodal tDCS protocols [13]. Work in animal models has established some initial knowledge regarding the mechanisms of NIBS in stroke rehabilitation. TMS displays a neuroprotective role for signaling molecules involved in the blood–brain barrier integrity and induces beneficial changes in angiogenesis [14], it modulates GABAergic interneuron transmission [15], as well as increasing expression of BDNF (brain-derived neurotrophic factor) [12,16]. Anodal tDCS of the contralesional hemisphere has been found to enhance perilesional neurogenesis in MCA (middle cerebral artery) occlusion, and also enhance expression of factors involved in downstream plasticity pathways [17,18]. NIBS has been applied in stroke patients mainly in the primary motor cortex and has shown greater efficacy combined with complementary motor practice techniques.

### 1.4. Limitations of NIBS—New Perspectives

Thanks to spontaneous neuroplasticity, the central nervous system (CNS) compensates in a varying degree for the functional disorder cause by stroke. Despite that, spontaneous reorganization may cause maladaptive plasticity, or prove insufficient for the retrieval of lost functionality [18]. The same principle underlies the use of non-invasive brain stimulation. For example, tDCS of the lower limb motor area may affect the muscles of both lower limbs, resulting in co-contraction [19]. Despite promising results from the application of NIBS techniques in stroke recovery, several gold-standard randomized clinical trials have procured negative results [18]. Wessel et al. (2018) collected data from studies concerning NIBS application following stroke and gathered potential factors that influence its response [20]. These include lesion site and size, the structural integrity of white matter tracts, time since stroke and level of impairment, stimulation site, mode, or duration [20]. For example, in a study by Ameli et al. (2009), 10 Hz rTMS applied to the ipsilesional M1 improved movement kinematics in patients with subcortical stroke, but not in patients who had also cortical stroke [21]. In a study by Lindenberg et al. (2012), patients with better corticospinal tract integrity showed a greater improvement of motor function after sessions of bihemispheric tDCS [16,22]. It has been shown that in cases of important brain lesion, the clinical efficacy of tDCS is affected by the metabolic cataract and loss of gray matter [23], while the development of porencephaly and the obstruction of current transmission are additional factors testing the effectiveness of cortical stimulation [24]. The lack of complete understanding of stroke physiology and plasticity mechanisms tests the effectiveness of cortical stimulation, seeing for example how the interhemispheric competition model cannot in all cases represent the changes following stroke [11].

In many cases, the motor deficits arising from a supratentorial stroke are associated with the phenomenon of crossed cerebellar diaschisis (CCD), which includes reduced blood perfusion and loss of spontaneous Purkinje cell firing [25]. CCD concerns a direct or indirect injury in the corticocerebellar tracts and is an indicator of clinical deterioration [20]. Further neuroplastic changes identified in the corticocerebellar system following stroke have shed light on the properties of cerebellum as a potential target for functional recovery. These include the cerebellar brain inhibition phenomenon (CBI), which refers to the natural tonic inhibition of the cerebral cortex by the cerebellum, and the disruption of learning mechanisms [20]. CBI is an estimate of cerebellocortical connectivity [26] and is largely affected by LTD, since it is found reduced after a period of adaptive learning [27]. The cerebellar brain inhibition phenomenon threatens the structural integrity of the cerebellothalamocortical tracts, and is associated with poor motor performance in chronic stroke. In various studies CBI was found reduced in patients who experienced a vascular lesion in the cerebellum, the thalamus, and the internal capsule [20]. According to the revised model by Doyon and Ungerleider (2005) concerning cerebral plasticity in the corticostriatal and corticocerebellar systems during learning of a new motor sequence or motor adaptation, the same structures are employed in the early period of learning, and interactions between those structures are crucial in establishing motor patterns necessary for skilled motor performance [20]. Neural representation of a new motor skill is thought to be distributed in a network of structures involving the corticostriatal or corticocerebellar circuit, depending on the type of motor learning needed [20]. It appears that the cerebral activation pattern following stroke resembles the one formed during motor skill learning [20]. Following supratentorial stroke in particular, cerebellum, remaining structurally integral, can offer an intervention passage to the motor learning network. Besides its remote position relative to the lesion, its compact structure makes it possible for anatomically small districts to send diffuse connections to the cerebral cortex [24]. According to a recent meta-analysis from Oldratti and Schutter (2018), cerebellar anodal and cathodal tDCS are proven to be efficient in modulating motor performance in healthy subjects in motor adaptation and motor skill learning tasks [28]. A recent systematic review by Kumari et al. (2019) attempts to collect experimental findings regarding the effect of cerebellar tDCS in motor learning in different time scales, in order to establish whether recorded benefits are sustained long-term after practice. The basic finding was a positive effect of anodal tDCS in facilitating motor skill learning in a short- (<24 h) and long-term (>24 h) level [29]. Balancing CCD, modulating CBI, and facilitating motor learning mechanisms are revealed to be both neurophysiological targets and strategies engaging cerebellar stimulation in stroke recovery [20].

The cerebellum plays a crucial role in various motor and non-motor functions, and especially regulating motor and non-motor behavior through adaptation and constant update of internal models, which facilitate error-based learning and motor control. Synaptic plasticity through LTD and LTP plays an important role in this process, by regulating Purkinje cell-parallel fiber synapses firing and consequently the cerebellar signaling to the cerebral cortex [30]. In this context, the cerebellum maintains a complex relationship with the brainstem, basal ganglia, and the cerebral cortex [31] that can be disrupted following cerebellar or cortical lesion through direct or indirect damage to the corticocerebellar tracts, and counts as a functional impact index depending on the underlying disorder. It is thus made clear that the cerebellum can raise to an intervention goal for the restoration of this relationship, and consequently for functional recovery.

Various studies have been published regarding the functional influence of cerebellar transcranial magnetic stimulation or direct current stimulation on motor cortex. Modulation of cerebellocortical connectivity depends on the different protocols used, as well as the interpersonal differences concerning circuitry and neurotransmission. A systematic review by Tremblay et al. (2016) attempts to collect experimental findings regarding NICS (non-invasive cerebellar stimulation) effect on different neurophysiological components of motor cortex neuroplasticity in healthy subjects and neurological patients, by using low- and high-frequency rTMS, iTBS or cTBS, tDCS, and PAS (paired-associative stimulation). The main study findings are the decreased CBI following cerebellar rTMS/tDCS, and the neuroplastic changes on M1 following PAS [32].

The field of cerebellar neuromodulation is rapidly evolving in the 21st century, both on an experimental and therapeutic level, in consistence with the advanced knowledge concerning cerebellar function in health and disease. The cerebellum has become a crucial neuromodulation target due to the high density of neural cells, the electric properties it possesses, and its involvement in various motor, cognitive, and affective loops [8]. So far, limited small-sample studies applying NICS in the rehabilitation of supratentorial stroke have been published, but to our knowledge based on literature search, no review regarding this intervention has been made. Consequently, the aim of this review is to explore the role of the cerebellum in motor and cognitive rehabilitation following cerebral stroke using NIBS techniques combined with other therapies (e.g., speech or physical therapy).

## 2. Materials and Methods

For this review, a systematic search of the literature was conducted in the databases PubMed and Scopus from July to October 2022 to include studies published between 2010 and 2022, by using the following keywords independently or combined by advanced search strategy: cerebellar stimulation, cerebellar TMS, cerebellar tDCS, stroke recovery (The advanced search included the combination of the keywords “cerebellar stimulation”, “cerebellar TMS”, “cerebellar tDCS” separately with the keyword “stroke recovery”, e.g., “cerebellar stimulation”, “cerebellar stimulation AND stroke recovery”).

Studies that met the following criteria were considered for inclusion in the present review: (a) they were classified as randomized clinical trials (RCTs); (b) participants suffered from cerebral stroke; (c) NIBS were under investigation; (d) both pre and post intervention data were presented. Studies were excluded according to the following criteria: (a) other study designs (review, meta-analysis, observational studies, case studies, etc.); (b) study protocols; (c) conference abstracts; (d) studies performed in animals; € reports not published in English; (f) retracted papers; (g) articles assessing different interventions (not NIBS) as well as (h) studies that also included participants with other neurological conditions (e.g., traumatic brain injury); (i) studies with participants without stroke or with cerebellar stroke; (j) studies without a control/placebo group or with healthy control group. Retrieved abstracts were meticulously assessed. In case of inability to establish if a study met the inclusion criteria, we reviewed the full text.

The following data were extracted according to standardized data extraction forms, and qualitatively analyzed: author, year of publication, number of participants, TBI stage, interventions assessed, cognitive status, cognitive domains assessed, study design, outcomes measures, duration and frequency of the intervention, results. Two independent reviewers (Ε.-A.Ν. and G.N.) conducted the literature search and data extraction independently. Discrepancies were resolved by a third author (A.Ν.).

## 3. Results

### 3.1. Study Selection

The extensive literature search provided 6681 results (PubMed, 4506; Scopus, 2175), which after the removal of duplicates were limited to 2370. Following title assessment, 1838 studies were excluded, while following further evaluation with abstract assessment 434 studies were not considered relevant for inclusion in this review. A total of 98 full texts were analyzed, and ultimately 28 clinical studies specifically concerning the use of NICS in stroke rehabilitation were assessed for eligibility for inclusion in this review. Seven studies were excluded based on the site of lesion (cerebellar stroke), two studies were case reports, and in four studies, full text or abstract was unavailable. Consequently, 15 randomized clinical trials (RCTs) were included for analysis. A total of 8 studies concerned the use of TMS protocols, in particular rTMS, iTBS, and PAS, and 7 studies concerned the use of tDCS. In total, 13 studies investigated aspects of motor recovery following cerebral stroke, and 2 studies investigated language recovery. Below are presented the findings of the RCTs based on the stimulation method applied and the functional target of recovery. The flowchart of the literature search is presented in Figure S1.

### 3.2. Cerebellar TMS in Stroke Rehabilitation

Applications regarding cerebellar TMS in cerebral stroke rehabilitation according to current literature concern dysphagia, gait-balance, and limb spasticity as outcomes of interest. Both rTMS and TBS have been studied but there is a prominent application of TBS, the main reason being that it can induce long-lasting cortical excitability with both lower stimulation intensity and training duration [33].

Initial findings concerning cerebellar TMS efficacy in rehabilitation of dysphagia arose from a study by Jayasekeran et al. (2011), where the interaction between the cerebellum and cortical activity in the pharyngeal representation area was investigated using TMS in healthy subjects, with an enhancement of pharyngeal motor evoked potentials (PMEPs) and increase in amplitude observed following paired pulse cerebellar TMS [34]. Further findings were shown by Sasegbon et al. in two consecutive studies, with bilateral cerebellar rTMS reversing inhibitory PMEPs induced by a ‘hemispheric stroke model’ [35,36]. Multiple fMRI studies have demonstrated the active role of the cerebellum in the deglutition process, and along with findings from TMS studies, it was deduced that the cerebellum is an important station of the swallowing network and can prove a key target in dysphagia rehabilitation [37].

The efficacy of an rTMS protocol was investigated by Zhong et al. (2021) in 143 patients with subacute stroke and certified dysphagia, with an important as well as similar improvement of dysphagia recorded in 2- and 4-weeks following intervention in all intervention groups [38]. The study by Rao et al. (2022) investigated an iTBS protocol [39] in 70 patients with endoscopically confirmed dysphagia. A clinically significant difference in improvement was recorded in favor of the real-iTBS group, with the degree of improvement approaching clinically the difference between nasogastric tube and oral feeding, or parenteral fluid supplementation vs. oral fluid intake [33].

Balance is considered an aspect of postural adaptation, and cerebellar hemispheres play an important part in motor adaptation. Improved balance is associated with improved gait performance, reduced risk of falls, and overall improved functional independency following stroke [39].

In the study of Koch et al. (2018), 36 chronic stroke patients with hemiparesis and balance disorder were submitted in daily sessions of either contralesional cerebellar iTBS, or sham iTBS supplemented with physiotherapy. Study findings included an overall increase in the Berg Balance Scale (BBS) score, which was clinically presented with a transition from supported to independent gait with reduced risk of falls, as well as a narrowing of stepping amplitude reflecting a steadier gait in participants who received iTBS, as well as enhanced neural activity in the posterior parietal cortex of the lesioned hemisphere [40]. In the study by Liao et al. (2020), balance and motor recovery in 30 hemiparetic stroke patients presenting with balance disorder (<56 score in BBS) were also assessed following the same iTBS protocol, the main study finding being an important between-group difference in favor of the intervention group in BBS score improvement following the intervention [39]. Similar findings arise from the study by Xie et al. (2021), which include a statistically important difference in favor of the intervention group recorded in the 10 MWT regarding gait performance [41].

Spasticity, a velocity-dependent elevation of muscle tone, is one of the most common debilitating findings in stroke survivors. Considering the role cerebellum exerts over motor control, along with positive findings from the use of cerebellar TBS in motor disorders such as levodopa-induced dyskinesia and cervical dystonia, cerebellar TBS has emerged as a promising experimental protocol for spasticity alleviation.

In a study by Dawei Li et al. (2021), three different TMS protocols were compared regarding their effects in muscle spasticity and limb dyskinesia in 90 chronic stroke patients (see Table 1) who also received physiotherapy or acupuncture treatment. The MAS (muscle tone test) score was significantly lower after treatment in all intervention groups, but more so in the combined intervention group, while the Fugl–Meyer and activity of daily living (ADL) assessment showed also marked improvement in the combined intervention group [42]. Rosso et al. (2022) studied a different protocol using paired-associative stimulation (PAS) between the contralesional cerebellum and the ipsilesional motor cortex in 27 chronic stroke patients with upper limb motor dysfunction. PAS is based on a classical conditioning-test TMS paradigm and is able to induce spike-time-dependent plasticity changes between two nodes [43]. A score-specific clinical improvement was recorded 30 days following the intervention in the active stimulation group in the Jebsen Taylor Test (JTT) score evaluating hand coordination and dexterity, and an equivalent increase in M1 activation was observed. It is thus concluded by the authors that the cerebellar-motor PAS exerts a plasticity-mediated positive effect in upper limb recovery in stroke patients, which may specifically favor cerebellar participation in motor functions [43]. Chen et al. (2021) studied an iTBS protocol to investigate the effects on upper limb poststroke spasticity in 32 subacute stroke survivors. A significant decrease in the modified Ashworth scale (MAS), the modified Tardieu scale (MTS), and upper limb elastography values were noted in the iTBS group compared to sham, leading to the conclusion that ipsilesional cerebellar iTBS can reinforce physical therapy gains in upper limp poststroke spasticity. The possible mechanisms include, according to the authors, the induced plasticity in Purkinje-cell level, as well as the influence on spinal neurons responsible for muscle tone adjustments [44]. Clinical studies concerning cerebellar TMS applications in stroke rehabilitation are presented in Table 1.

### 3.3. Cerebellar tDCS in Stroke Rehabilitation

Cerebellar tDCS has been studied in post-stroke gait-balance disorders and aphasia. In a randomized clinical trial by Zandvliet et al. (2018), 15 patients with chronic supratentorial stroke and standing balance disorder (<56 score in BBS), and 10 healthy age-matched controls were submitted in an anodal cerebellar tDCS protocol during a postural tracking task. Standing balance performance was evaluated in three static positions, with open or closed eyes, and in a tandem position, with an important decrease in center of pressure (CoP) parameters recorded in the more demanding tandem position in patients who received contralesional anodal stimulation [45]. Ranjan et al. (2021) investigated the effects of lobule-specific cerebellar tDCS on postural control in 12 chronic stroke subjects with variable lesion areas (e.g., lateral cortex, basal ganglia), who were submitted to two ctDCS montages concerning the dentate nuclei and the lobules VII-IX, respectively, pro and post a task of weight-shifting in a VR-based platform. Study findings can be summarized in differentiated findings of the two montages regarding kinetic measures, with the dentate nuclei stimulation exerting a more important effect on postural control, and the lobule VII-IX stimulation exerting an inhibitory effect on the dentate nuclei. Furthermore, non-responders with basal ganglia infarction demonstrated collectively poor postural control [19]. In a similar protocol study, Solanki et al. (2021) investigated the lobule-specific electric field effects of cerebellar tDCS on overground gait performance in 10 chronic stroke patients, by applying two distinct bilateral ctDCS montages targeting the dentate nuclei and the lower-limb representation lobules (VIIb-IX). The dentate nuclei montage was found to affect dentate nuclei as well as posterior and anterior lobules in terms of electric field distribution, while the leg montage affected the posterior lobules and the dentate nuclei. The positive effects of the two montages were similar during clinical assessments, while the mean lobular electric field strength was positively correlated with gait parameters procured from a gait quantification shoe, such as ‘Step time affected leg’ and ‘Stance time unaffected leg’. Findings from this preliminary study suggest that the ‘amount’ of the electric field created plays an important role in improvement of performance [3].

Based on the positive findings of a previous study concerning the enhanced efficacy of robotic therapy in chronic stroke patients after application of cerebral tDCS combined with transcutaneous spinal direct current stimulation (tsDCS), Picelli et al. (2018) conducted a new study of a similar protocol, applying this time cerebellar tDCS in 20 chronic stroke patients. The theoretical framework lies on the neuronal basis of gait generation and control, which consists of complex control mechanisms including supraspinal structures and spinal centers that form the central pattern generators, cerebellum being an important node of this network [46]. The authors aimed in this study to compare two different strategies for reestablishing inter-hemispheric balance and reversing transcallosal inhibition, either by directly stimulating the lesioned M1 with ipsilesional cerebral tDCS, or by indirectly stimulating the contralesional cerebellothalamocortical tract with contralesional cerebellar tDCS [46]. The main finding was an important difference in gait performance improvement as assessed by the 6 MWT in the group receiving cerebellar tDCS directly following intervention, with similar findings arising from the gait pacing analysis. Furthermore, patients who received cerebellar stimulation demonstrated a substantial improvement in affected limb motility in all timescales of evaluation. Findings support the authors’ assumption that the second strategy may prove more efficient in enhancing robotic therapy gains combined with tsDCS, a notion that could be attributed to the benefits of stimulating a structurally and functionally integral area [46]. In the same pattern, Picelli et al. (2019) conducted a new study with 40 chronic stroke participants, which aimed once more to compare two different strategies (contralesional/ipsilesional tDCS) for enhancing functional recovery based on two different theories, the first assigning to the healthy cerebral hemisphere a harmful role for functional recovery, due to increased transcallosal inhibition, and the second one attributing a positive role to the healthy hemisphere, based on the hypothesis of functional reorganization. Overall, an important improvement was recorded for each group in gait and mobility parameters evaluated also in the previous study, with authors suggesting a possible involvement of both theories in reorganization mechanisms in this particular patient’s sample [47].

The potential uses of non-invasive cerebellar stimulation in aphasia rehabilitation have emerged through findings concerning cerebellar involvement in non-motor aspects of language, such as phonetic and semantic fluency [24]. Increased activation of right cerebellar hemisphere is associated with improved performance in aphasic patients [24], while in chronic aphasic patients a loss of cerebellar gray matter has been observed, associated with reduced speech production [24,48]. Important findings establishing a beneficial effect of cerebellar tDCS in aphasia rehabilitation came from a study by Turkeltaub et al. (2016), where an improved performance in phonemic fluency following tDCS in posteriolateral cerebellum, noted at a higher degree with anodal stimulation, was recorded in healthy participants, while at the same time increased connectivity between cerebellum and cortical language networks concerning both motor and non-motor language aspects was observed. The authors suggested three positive effects of cerebellar tDCS in aphasia rehabilitation, direct improvement of speech performance, modulation of language networks in the left cerebral hemisphere, and facilitation of language learning mechanisms [24]. Marangolo et al. (2018) investigated the efficacy of cerebellar tDCS in enhancing language therapy gains in 12 patients with chronic left cerebral hemisphere stroke presenting with mild non-fluent aphasia during a verb generation task and during a verbal naming task. Following intervention, an improvement in mnemonic verb retrieval was observed in the verb generation task [49]. In a study by Sebastian et al. (2020), right cerebellar tDCS combined with computer-based language therapy was evaluated in 21 aphasic patients. Language performance was assessed through measurements in two tasks of trained and untrained naming, with a better efficacy of the tDCS recorded in both tasks, enhanced two months following the intervention [50]. Clinical studies concerning cerebellar and tDCS applications in stroke rehabilitation are presented in Table 2.

## 4. Discussion

The present review is the first one to focus on the role of the cerebellum in motor and cognitive rehabilitation after cerebral stroke using NIBS techniques combined with other therapies (e.g., speech or physical therapy). Findings arising from published RCTs raise a promising perspective regarding the use of non-invasive cerebellar stimulation following cerebral stroke. In the motor domain, improvement in performance is recorded in patients suffering from dysphagia, gait, or balance disorders and limb spasticity/dyskinesia. Language recovery findings are only preliminary with recorded improvement in verb naming and retrieval. Most studies are consistent in showing improvement in the clinical question, with differences in improvement and efficacy degree rising from protocol and stimulation techniques variability, as well as interindividual differences regarding stroke severity or mechanisms of recovery. Gains in performance are recorded in all studies following a combined intervention of non-invasive stimulation and physical/speech therapy or newer rehabilitative techniques such as robotic therapy.

Regarding the functional influence of non-invasive cerebellar stimulation on motor cortex, in general it has been shown to induce plasticity mechanisms at the cortical level and consequently at the cerebellocortical networks, demonstrated both by enhanced connectivity, and by modulation of MEP’s amplitudes and CBI [51]. Part of this functional influence lies, reversely, in certain neurophysiologic phenomena related to stroke which affect the corticocerebellar system increasing the lesion extent, the most important being the crossed cerebellar diaschisis phenomenon (CCD), the cerebellar brain inhibition (CBI) dysfunction, and the disorder of learning procedures [16]. Moreover, cerebellar activity, assessed through structural and functional connectivity, is associated both with motor recovery and the residual motor output following supratentorial stroke [51]. Neurophysiological measurements from the studies included in this review did not show a significant increase in MEP amplitudes in the affected hemisphere following non-invasive cerebellar stimulation, regardless of the clinical improvement observed [39,41,42,43,44].

The concept of cerebellar stimulation engages, according to the current literature, both the interhemispheric inhibition model and the vicariation model for stroke plasticity. For example, cathodal stimulation of the contralesional cerebellar hemisphere improves gait performance compared to anodal ipsilesional cortical stimulation in the study by Picelli et al. (2018), by indirectly enhancing the cerebellothalamocortical pathway and reversing CBI, but the same protocol did not show a predominance compared with cathodal ipsilesional cerebellar stimulation, which in turn enhances the activity of the contralesional cerebral hemisphere, in the following study [47].

The parameters of the cerebellar s”Imu’ation were defined mostly based on data from previous studies, which employed MRI reconstruction and neuronavigation techniques. Regarding tDCS, the electrodes were applied in the cerebellum according to the international 10/20 or 10/5 EEG systems, with current intensity either at 1.5 mA or 2 mA. In the studies concerning TMS, a figure-of-eight coil was applied tangentially to the skull in a position defined by previous studies as optimal for eliciting MEPs of the highest amplitude in the cortical region of interest. For example, in studies investigating dysphagia recovery, the coil was positioned 4.3 cm lateral and 2.4 cm below the inion, an area corresponding to the pharyngeal cortical representation area [38], while in studies investigating gait/balance recovery, the coil was positioned 3 cm lateral and 1 cm below the inion, a site which targets the posterior and superior cerebellar lobules [42]. The stimulus intensity was set to 80–110% of resting motor threshold (RMT) or 80% of active motor threshold (AMT), with concerns arising about efficacy based on the fact that the RMT in the affected hemisphere is generally higher and demands higher stimulation intensity, which can cause patient discomfort [43]. There were no serious safety issues met in the included studies.

Cathodal tDCS of the contralesional cerebellar hemisphere and iTBS of the contralesional cerebellar hemisphere show the most frequent application in published studies concerning gait/balance rehabilitation, with recorded efficacy related to the facilitation of the lesioned cerebral hemisphere. A comparison of efficacy of applied protocols concerning limb spasticity or dysphagia cannot be made based on included studies, as there is protocol variability, but in both cases, TMS protocols have been applied and proven efficient. It is confirmed that motor output concerns the function of a multimodal network of structures, with stimulation of a network node affecting further network areas [40]. Reorganization patterns and neurophysiological changes, along with the residual motor output, determine each protocol’s efficacy. tDCS advantages over TMS techniques include the wider anatomical effect, greater flexibility, and safety in use, as well as easier combination with supplementary rehabilitative techniques in clinical practice. iTBS offers the advantages of the short application duration, the long-term effects achieved with lower-intensity stimulation, and the induction of synaptic plasticity [41]. It has been proposed that cerebellar TBS exerts a modulatory effect on thalamic or cortical interneurons dependent on GABA activity, which is crucial for plasticity-driven mechanisms in stroke recovery [40].

Findings concerning the use of non-invasive cerebellar stimulation in aphasia rehabilitation following stroke remain limited, and arise from limited-population studies, with tDCS proving a more favorable technique. There is no obvious superiority between anodal/cathodal stimulation, possibly due to the complexity of the cerebellar cortical folding which may cause simultaneous depolarization and repolarization of neurons, and lead to different overall effects, while subjective anatomical and neurophysiological parameters play an important part in different polarity effects [50]. Marangolo et al. (2018) attribute right cerebellar cathodal tDCS efficacy to the activation of left frontal areas through disinhibition of Purkinje cells, an assumption supported by study findings showing similar facilitating patterns in verbal fluency tasks by cathodal cerebellar stimulation or anodal frontal lobe stimulation [49]. Findings from published studies show a positive effect of cerebellar stimulation in various linguistic domains, although detailed analysis demonstrates heterogeneity between studies. This could be related to the fact that different treatment strategies exert different effects in plasticity mechanisms inside language networks and can involve networks of both left and right hemisphere [50]. Furthermore, non-linguistic parameters are involved in the process of reforming neural networks that support language recovery in aphasic patients, which may also define functional severity [52]. It appears that cerebellar involvement in language process is strongly associated with cognitive demands, through release of left prefrontal cortex cognitive resources, and engagement of processes such as working memory and executive functions [49].

Plasticity mechanisms in the corticocerebellar system following cerebral stroke, and findings regarding cerebellar involvement in gait and balance control, language, and cognitive networks, render the cerebellum an important neuromodulation target. Different study and stimulation protocols, small clinical samples, and subjective differences in circuitry and stroke semiology prevent the extraction of safe conclusions regarding the use of NICS in stroke rehabilitation, but study findings open encouraging perspectives for establishing its use. Factors affecting its efficacy, but also setting its possible advantage over cortical stimulation, include the location and size of stroke lesion affecting current transmission, the white matter tracts structural integrity, the degree of disorder, the residual functionality, the time passed from stroke appearance, and the location, protocol, and duration of stimulation [20].

## 5. Limitations and Future Studies

The main limitations of this review are the small number of clinical studies published so far, as well as the small clinical sample included. Furthermore, the variability of NICS techniques and the non-established consensus on the investigated protocols render the interpretation of findings and the comparison between protocols challenging. Most studies lacked an individualized approach concerning the side of stimulation, while stimulus or current intensity varied between studies. In addition, some studies lacked long-term follow-up so lasting effects of the stimulation cannot be yet observed. Moreover, not all studies included neurophysiological measurements that could interpret clinical and functional outcomes and could provide prognostic values, while in the studies that included neurophysiological measurements, the results did not reflect the clinical outcome. Current results regarding NICS in stroke recovery have a proof-of-concept value based on published studies. Although a direct comparison between cerebellar and cortical stimulation cannot be made in the context of stroke recovery based on current literature, it is made clear that cerebellar stimulation can provide therapeutic alternatives that can prove useful depending on the functional target, the lesion site/severity, and the individual mechanisms affecting plasticity.

The main challenge of cerebellar stimulation lies in the question of its future response to the perspectives it has established according to current findings. Future studies are expected to include a larger population sample for better reproducibility and credibility of various findings. In addition, further customization of NICS protocols regarding stroke rehabilitation will enable better comparison between studies. This might be assisted by the development of mathematical models that will predict current diffusion and distribution and will increase stimulation precision [37], as well as advanced neuroimaging modalities that will assess neurophysiological values. Rezaee et al. (2021) showed the feasibility of a combined functional near-infrared spectroscopy (fNIRS) and electroencephalography (EEG) protocol to measure changes in cortical activation that could predict response to cerebellar tDCS treatment in chronic stroke survivors with hemiparesis [53]. Areas of interest for future studies could include the simultaneous stimulation of different brain areas targeting different stations of involved networks, or the combination of central and peripheral stimulation, the individualized approach concerning stimulation position using advanced neuroimaging, and the introduction of protocols that will encompass supplementary rehabilitation techniques aiming in improving performance both short- and long-term.

## 6. Conclusions

Despite encouraging results, long-term effects of cerebellar stimulation in sustaining functional gain need to be recorded and evaluated considering other factors affecting rehabilitation. Moreover, policies that will assist the transfer of arising findings from experimental to wide clinical practice need to be implemented, taking into consideration the patients’ benefit.

Overall, establishment of stroke as a main short- and long-term functional disability source renders necessary the application of new rehabilitation protocols that will respond to its broad semiology and varying functional impact. The cerebellum can prove a crucial intervention target in rehabilitation following stroke. Non-invasive cerebellar stimulation appears to be a promising tool in this context, and its use in clinical practice remains to be established in the direct future.

## Figures and Tables

**Table 1:** Clinical studies concerning cerebellar TMS applications in stroke rehabilitation.

Author/Year	NICS	Sample	NICS Protocol	Supplementary Technique	Functional Outcome	Main Results
Zhong et al., 2021	Cerebellar rTMS	Subacute stroke n = 38 lesioned hemisphere intervention group n = 39 healthy hemisphere intervention group n = 35 cerebellum intervention group n = 35 control group	20 min. 5 Hz rTMS for 2 w. (10 sessions) in the mylohyoid cortical region	Traditional dysphagia treatment (e.g., vocal cord/oropharyngeal exercises)	Dysphagia	Dysphagia improvement equal in all groups
Rao et al., 2022	Cerebellar iTBS	Acute/Subacute/chronic stroke n = 35 intervention group n = 35 sham control group	3 pulses of 50 Hz/200 ms with 8 s interval-total of 600 pulses to each hemisphere (10 sessions)	Traditional dysphagia treatment (e.g., vocal cord/oropharyngeal exercises)	Dysphagia	Greater improvement of dysphagia in real iTBS group
Koch et al., 2018	Cerebellar iTBS	Chronic stroke n = 18 intervention group n = 18 sham control group	2 pulses with a 5 s time interval, sum of 1200 pulses to contralesional hemisphere (15 sessions)	Physical therapy	Gait-balance	Improved gait stability, transition in independent gait in real iTBS group
Xie et al., 2021	Cerebellar iTBS	Chronic stroke n = 18 intervention group n = 18 sham control group	3 pulses of 50 Hz/200 ms with 8 s interval-total of 600 pulses to contralesional hemisphere (10 sessions)	Physical therapy	Gait	Greater improvement of 10 MWT in real iTBS group
Liao et al. 2020	Cerebellar iTBS	Subacute/Chronic stroke n = 15 intervention group n = 15 control group	total of 600 pulses to contralesional hemisphere (10 sessions)	Physical therapy	Balance	Improved BBS score in real iTBS group
Dawei Li et al., 2021	Cortical rTMS+ Cerebellar cTBS	Chronic MCA stroke n= 30 unaffected M1 lf-rTMS+ right cb. cTBS intervention group n = 30 M1 lf-rTMS intervention group n = 30 right cb. cTBS intervention group	20 min. 1 Hz rTMS in the unaffected M1 3-pulse bursts at 50 Hz right cerebellar cTBS (24 sessions)	Physical therapy/acupuncture	Muscle spasticity Limb dyskinesia	Improvement of spasticity higher in combined intervention group
Chen et al., 2021	Cerebellar iTBS	Subacute stroke n = 16 intervention group n = 16 sham control group	total of 600 pulses to ipsilesional hemisphere (10 sessions)	Physical therapy	Upper limb spasticity	Improvement of spasticity higher in intervention group
Rosso et al., 2022	Cerebello-motor PAS	Chronic stroke n = 14 intervention group n = 13 sham control group	Active stimulation: Conditioning stimulus over contralesional cerebellum Test stimulus over ipsilesional M1 Sham stimulation: Sham stimulus over cerebellum Test stimulus over M1 (5 sessions)	Physical therapy	Upper limb motor recovery	Clinical improvement in the JTT score for hand coordination and dexterity, ↑ M1 activation in the active stimulation group

Abbreviations: BBS: Berg balance scale; cTBS: continuous theta-burst stimulation; iTBS: intermittent theta-burst stimulation; JTT: Jebsen–Taylor hand function Test; lf-rTMS: low-frequency rTMS; M1: primary motor cortex; MCA: middle cerebral artery; 10-MWT: 10-min walking test; PAS: paired-associative stimulation; rTMS: repeated transcranial magnetic stimulation.

**Table 2:** Clinical studies concerning cerebellar tDCS applications in stroke rehabilitation.

Author/Year	NICS	Sample	NICS Protocol	Supplementary Technique	Functional Outcome	Main Results
Zandvliet et al., 2018	Cerebellar tDCS	Chronic stroke n = 15 patients intervention group n = 10 healthy control group	20 min. 1.5 mA, anodal contralesional/ ipsilesional cerebellar stimulation/sham stimulation (3 sessions)	None	Standing balance	Improved standing balance with contralesional cerebellar tDCS in tandem position
Picelli et al., 2018	Cerebellar tDCS + transcutaneous spinal direct current stimulation (tsDCS)	Chronic stroke n = 10 intervention group A n = 10 intervention group B	A: cathodal contralesional cerebellar tDCS (20 min. 2 mA) Β: anodal ipsilesional cerebral tDCS (20 min. 2 mA) (10 sessions)	Robotic therapy	Gait	Difference in improvement in 6MWT performance right after intervention in group A Difference in improvement if affected limb mobility in group A
Picelli et al., 2019	Cerebellar tDCS+ tsDCS	Chronic stroke n = 20 intervention group A n = 20 intervention group B	A: cathodal contralesional cerebellar tDCS (20 min. 2 mA) B: cathodal ipsilesional cerebellar tDCS (20 min. 2 mA) (10 sessions)	Robotic therapy	Gait	Improvement in 6-MWT, limb motility for each group
Solanki et al., 2021	Cerebellar tDCS	Chronic stroke n = 10 patients Crossover study	2 bilateral tDCS (15 min 2 mA) montages: A: dentate nuclei B: lobules VIIb-IX (2 sessions)	None	Gait	Equal improvement in gait parameters (e.g., Step length, Stance time)—correlation with lobular electric field strength
Ranjan et al., 2021	Cerebellar tDCS	Chronic stroke n = 12 patients crossover study	2 bilateral tDCS (15 min 2 mA) montages: A: dentate nuclei B: lobules VII-IX (2 sessions)	None	Postural control	Positive effect of the dentate montage on postural control
Marangolo et al., 2018	Cerebellar tDCS	Chronic left hemisphere stroke, n = 12 patients, crossover study	20 min. 2 mA cathodal right cerebellar tDCS/sham stimulation (20 sessions total)	Speech therapy	Language recovery	Improved mnemonic verb retrieval
Sebastian et al., 2020	Cerebellar tDCS	Chronic left hemisphere stroke, n = 21 patients, crossover study	Phase A: 20 min. 2 mA anodal right cerebellar tDCS/sham stimulation Phase B: 20 min. 2 mA anodal right cerebellar tDCS/sham stimulation (15 sessions/phase)	Speech therapy	Language recovery	Improved performance in untrained picture naming task

Abbreviations: 6-MWT: 6-min walking test; mA: milliampere; tDCS: transcranial direct-current stimulation; tsDCS: transcutaneous spinal direct-current stimulation; VII-IX: cerebellar lobules VII-IX.

## Data Availability

Not applicable.

## Supplementary Materials

The following supporting information can be downloaded at: https://www.mdpi.com/article/10.3390/healthcare10122401/s1, Figure S1: Flowchart of the literature search of this review.
