# Supplementary figures and images for: Targeting Cerebellum with Non-Invasive Transcranial Magnetic or Current Stimulation after Cerebral Hemispheric Stroke—Insights for Corticocerebellar Network Reorganization: A Comprehensive Review
Source: Healthcare (Basel). 2022 Nov 29;10(12):2401. doi: 10.3390/healthcare10122401 (PMC9778071; doi:10.3390/healthcare10122401)

**Figure S1. Flowchart of the literature search of this review**

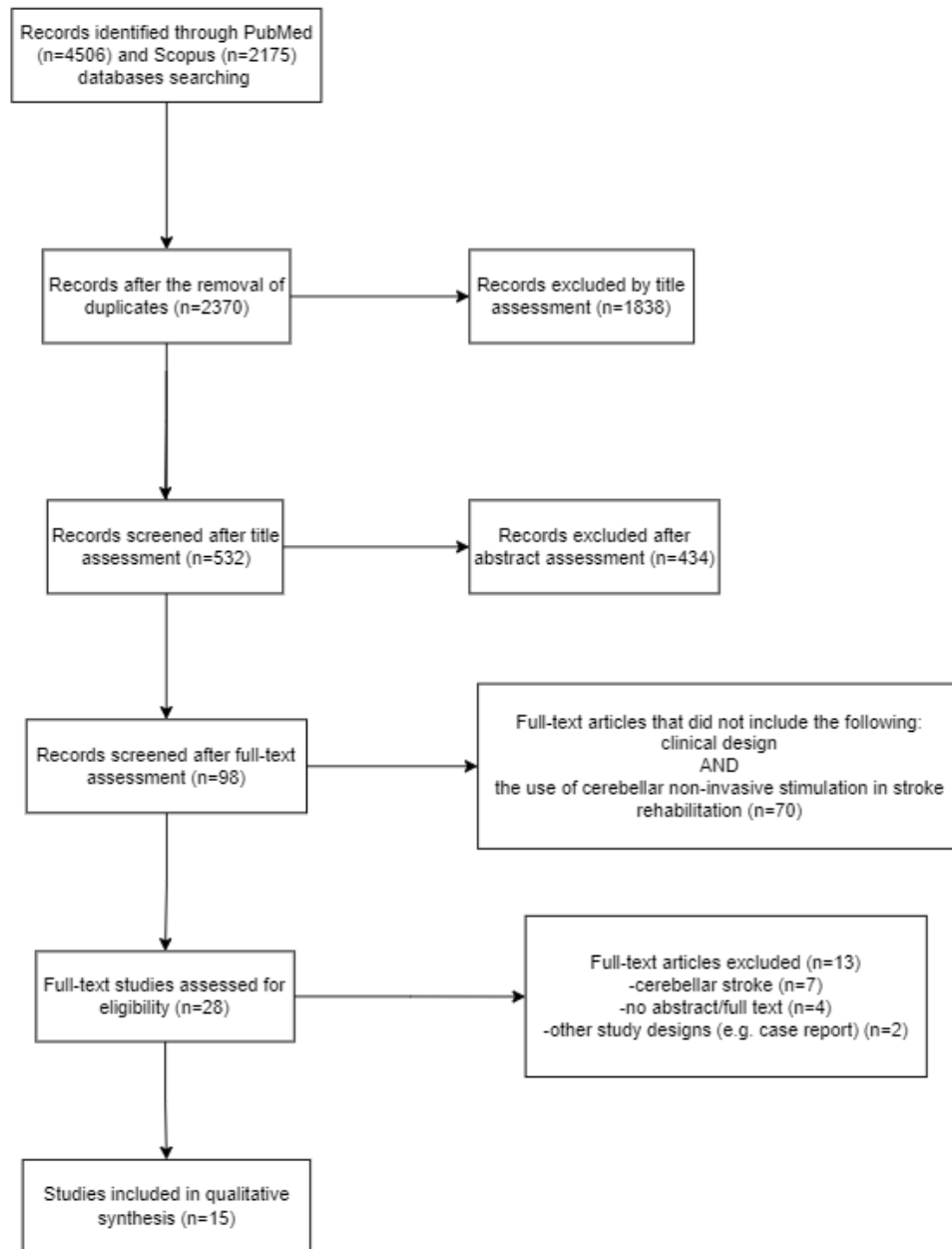

Supplement: Supplementary file 1 [file healthcare-10-02401-s001.zip › healthcare-2054490-supplementary.pdf]
